# Supplementary material for: Chromothripsis during telomere crisis is independent of NHEJ, and consistent with a replicative origin
Source: Genome Res. 2019 May;29(5):737–49. doi: 10.1101/gr.240705.118 (PMC6499312; doi:10.1101/gr.240705.118)
Supplement: Supplemental Material [file supp_gr.240705.118_Supplemental_file_1.zip › contigs/annotated_contigs/DB115/contig.2.DB115_length_766_mean_cov_10.3785900783.docx]

**DB115_length_766_mean_cov_10.3785900783**

```
CTTGTAGTGTTTCATCATCTCTTGTTGGGTTCATTTTATCCCGGCTCACACTTTGTCAATATTCCCTTAATTAACCTCTACTCAATCAA
 >chr4:44237604-44238016 - E=3e-231 p=3e-02
GAACTTTGAGTGTCTGTGTCTTGCCATGACCTTGACTGATACAGGGACTCATTGTAATCAAGGTGAAAGCTGTGCATAAAACTGAAGCC

TGCAATGTTTTGTAGTATGTTTGGGGGAAGATTGACTCATCACTCTTTAAACAAAAAGATGTGCTATATAATTGAGAGATATATGATAT

TATTTCTCTGTTAAATCCTATGTACTACTTAAAATAAGTTGGTCATGAATAAGATATTAAATAAAACTATTATTAGTAGACTGAAAGCT

GCTTTGTCAGTTTCTGCAGACAGTGGATTTTACTCATACTCTCTCTCTCTCTCTC|CACTGTCTCC|TTTTCCACTTTCCCACTCCAAC
                                                                  >chr4:44234625-442349
TTTAGCTCCTCAAAGACAACAATTTTCTACTTATTCACTAGTTTGTTTCATCATTTATGTCCACATTTCTAGATAATAAGCATCTAATA
69 - E=6e-191
CTAGTTTATTTAAATAATTAATTTAAAATAAGTATAAATAATATTGTAATGTACTTATATCTCAACTTAGAAATAAAACATAGCAGCTA

CTGTTGGATTGCTGTTTCCCCAAAGGAAACTATTATTCTGTTTGTTGTTTATGATTTTTTATTAAGCTAGCATTGTAATAGCTCAATCT

ATTTATTTTTGTCATATCTCAGAAGATAAAACTAATGCAAAGTTGATTCTTCTATT
```
